# A Refined Mobile Health Intervention (SMARTFAMILY2.0) to Promote Physical Activity and Healthy Eating in a Family Setting: Randomized Controlled Trial

**DOI:** 10.2196/65558

**Published:** 2025-12-15

**Authors:** Janis Fiedler, Kathrin Wunsch, Sebastian Hubenschmid, Harald Reiterer, Britta Renner, Alexander Woll

**Affiliations:** 1 Institute of Sports and Sports Science Karlsruhe Institute of Technology Karlsruhe, Baden-Wurttemberg Germany; 2 Fresenius University of Applied Sciences Heidelberg, Baden-Wurttemberg Germany; 3 Department of Computer and Information Science University of Konstanz Konstanz, Baden-Wurttemberg Germany; 4 Department of Psychology University of Konstanz Konstanz, Baden-Wurttemberg Germany

**Keywords:** mobile app, telemedicine, behavior change, health behavior, family, primary prevention, exercise, diet, food and nutrition, randomized controlled trial, accelerometer, wearable electronic devices, social-cognitive determinants, just-in-time adaptive intervention, digital intervention

## Abstract

**Background:**

Many mobile health (mHealth) apps focus on promoting physical activity (PA) and healthy eating (HE). However, there is limited empirical evidence regarding their effectiveness in initiating and sustaining behavior change, particularly among children and adolescents. Considering that behavior is influenced by social contexts, it is essential to take core settings like family dynamics into account when designing mHealth apps.

**Objective:**

The purpose of this study was to further develop and refine the *SMARTFAMILY* (SF) app targeting PA and HE in a collective family-based setting by enhancing design and usability, as well as by adding gamification aspects, health literacy, and just-in-time adaptive interventions to the first version of the app.

**Methods:**

The SF2.0 app, based on behavior change theories and techniques, was developed, implemented, and evaluated. The app was used in a collective family setting, with family members using it individually and cooperatively. In a cluster-randomized controlled trial, the intervention group (IG) used the app for 3 consecutive weeks, while the control group (CG) received no treatment. Primary outcomes included PA measured through self-reports and accelerometry, as well as self-reported fruit and vegetable intake (FVI) for HE. Secondary outcomes included intrinsic motivation, behavior-specific self-efficacy, and the Family Health Climate. A follow-up assessment (T_2_) was conducted 4 weeks after the postmeasurement (T_1_) to assess intervention effects. Multilevel analyses were performed in R (R Foundation for Statistical Computing), considering the hierarchical structure of individuals (level 1) within families (level 2).

**Results:**

Overall, 55 families (28 CG, 105/209; 27 IG, 104/209 participants) were recruited for the study. In total, 3 families (3 CG, n=12) chose to drop out of the study due to personal reasons before T_0_. Overall, no evidence for meaningful and statistically significant increases in PA was observed in favor of the IG of our physically active sample. However, the app elucidated positive effects in favor of the IG for FVI diary (T_0_-T_1_; *P*=.03), joint PA (T_0_-T_1_; *P*=.02 and T_0_-T_2_; *P*<.001), and joint family meals (T_0_-T_1_; *P*=.004).

**Conclusions:**

The SF2.0 trial evaluated an mHealth intervention designed to promote PA and HE within families. Despite incorporating a theoretical foundation, several behavior change techniques based on family life, and gamification and just-in-time adaptive intervention features, the intervention did not significantly increase PA levels among physically active participants. FVI, joint PA, and joint meals were improved within the IG. Previous studies on digital health interventions have produced mixed results, and family-based mHealth interventions remain rare, with limited focus on whole-family behavior and randomized controlled trials. To enhance intervention effectiveness, future app development could consider incorporating even more advanced features and should focus on inactive participants. Further research is needed to better understand intervention engagement and tailor mHealth approaches for primary prevention efforts.

**Trial Registration:**

German Clinical Trials Register DRKS00010415; https://www.drks.de/search/en/trial/DRKS00010415

**International Registered Report Identifier (IRRID):**

RR2-10.2196/20534

## Introduction

### Background

The health benefits of sufficient physical activity (PA), reduced sedentary behavior including screen time, and healthy eating (HE) are manifold [1-4] and include benefits regarding all-cause mortality, cardiovascular function, cancer, and metabolic and mental health, among others [3,5-7]. Hence, engaging in different health behaviors should be emphasized from a young age onward. Guideline adherence is only high for PA in preschool children [8], but the older the children get, it becomes less likely that they sufficiently engage in PA [9-11]. Adherence to screen time guidelines is generally low [12,13], and children are known to make frequently unhealthy food choices [14-17]. It has been observed that the majority of children and adolescents (81%) and a significant proportion of adults (23%) do not meet the recommended levels of PA and HE, specifically in terms of fruit and vegetable intake (FVI), globally [18]. Research has shown that there is a dose-response relationship, meaning that even small increases in PA or HE can have positive effects on the physiological and psychological health of children and adolescents [19] and adults [2,20-23].

A promising way to achieve sustainable behavior change is to create interventions that target children and adolescents, as longitudinal studies showed that behavioral patterns in childhood and adolescence have a low to moderate influence on PA patterns in adulthood [24-29]. As children and adolescents are strongly dedicated to siblings and parents, social contexts like the family context need to be involved in intervention development, as health behaviors are affected by social relations and ties [30,31]. By implementing the family context into the intervention design, HE [32] and PA [33,34] can be facilitated more sustainably by targeting variables of daily family life like common meals [35-37]. Results of intervention studies also indicate that social support is significantly associated with the continuation of exercise programs [38-42] as well as participation in weight-loss interventions [43-45]. Therefore, both parents and their children can benefit from a behavior change intervention at the family level.

To deliver such interventions and individualize them to the different needs of participants, mobile health (mHealth) interventions have become an increasingly popular strategy for promoting PA and HE through smartphone apps, text messaging, and wearable technologies. Smartphone-based apps in particular are promising to change health behavior and be cost-effective on a large scale [46-48]. These tools aim to improve health behaviors by providing real-time feedback, personalized goal setting, and behavior change support.

Meta-analyses and systematic reviews consistently show that mHealth interventions (including apps, SMS, and web-based tools) lead to significant improvements in both PA and diet quality across diverse populations [49-51]. Recent reviews and meta-analyses provide preliminary evidence for the effectiveness of app-based health behavior change [52-54]. Certain facets of health behavior change are related to intervention effectiveness [50], including a theoretical foundation and behavior change techniques (BCTs [55]) like goal setting, self-monitoring, and social support [56,57], implementing the intervention in a social context [30,58], and the tailoring of interventions to participants’ needs. However, while many existing apps effectively raise awareness and short-term motivation, sustained engagement and long-term behavior change remain challenging [50,59]. Most studies and apps focus on either PA or diet alone, but a significant and growing subset addresses both behaviors together, pointing to higher effectiveness in combined health behavior interventions [52,60-63].

Overall, there is a lack of randomized controlled mHealth studies targeting both children and adults within a social system. In addition, most studies are not aiming at changing more than 1 health behavior [64] and do not sufficiently use important key facets of effective interventions like BCTs. Moreover, many studies do not provide a theoretical foundation or incorporate just-in-time adaptive interventions (JITAIs) [65-67].

### Objective

Despite the growing number of mHealth apps promoting PA and HE, many interventions remain limited by a focus on individual users, generic feedback, a lack of BCTs, and a theoretical foundation. This study builds directly on the initial *SMARTFAMILY* (SF) intervention, which was designed as a family-based mHealth program to promote PA and HE through self-monitoring, goal setting, and feedback. Unlike most existing apps, SF2.0 (as well as SF) targets families as interactive units, fostering shared goals and mutual support between parents and children. SF2.0 retains this theoretical foundation but introduces several key innovations. First, SF2.0 integrates JITAI principles, allowing the app to deliver context-sensitive prompts, and feedback tailored to users’ activity patterns and daily routines. Second, gamification elements (eg, team challenges and family-based achievements) were added to strengthen intrinsic motivation and engagement. Third, based on insights from the initial SF feasibility trial, measurement instruments and app usability were refined, and an additional outcome—behavioral intention—was incorporated to better capture motivational processes. Furthermore, 3 more BCTs were added, and physical literacy aspects were incorporated to strengthen health literacy. By combining these adaptive, social, and motivational elements within a scientifically grounded framework, SF2.0 aims to generate sustained behavior change and provide empirical evidence on mechanisms that may enhance the effectiveness of digital health interventions. Thus, while SF2.0 replicates the core components of the original SF intervention to ensure comparability, it advances the digital behavior change design by testing whether these adaptive and motivational enhancements lead to stronger and more sustained effects on family health behavior.

Whereas the original SF app was already based on earlier mentioned principles (ie, was theory-based, used the social family setting, and included BCTs), the SF2.0 app was further developed based on current literature by revising the user interface and usability and by adding and consolidating several other features that have been shown to positively influence intervention effectiveness, like gamification [68], physical literacy [69,70], further BCTs [55], ecological momentary assessments (EMAs) [71], and JITAIs [72]. Recent developments in technology, mobile sensing, and wireless communication enable researchers to deliver JITAIs [65,72] at the most promising time for behavior change and adapt them in real time to sensor or participant input. The evidence for these interventions is preliminary but promising to maximize effectiveness while minimizing participant burden [73-75].

For more details and the complete study protocol, see Wunsch et al [76]. A positive influence of the refined mHealth intervention on PA variables steps and moderate to vigorous physical activity (MVPA) and the HE variable FVI in the whole family was hypothesized.

## Methods

### Study Design

**Figure 1:**
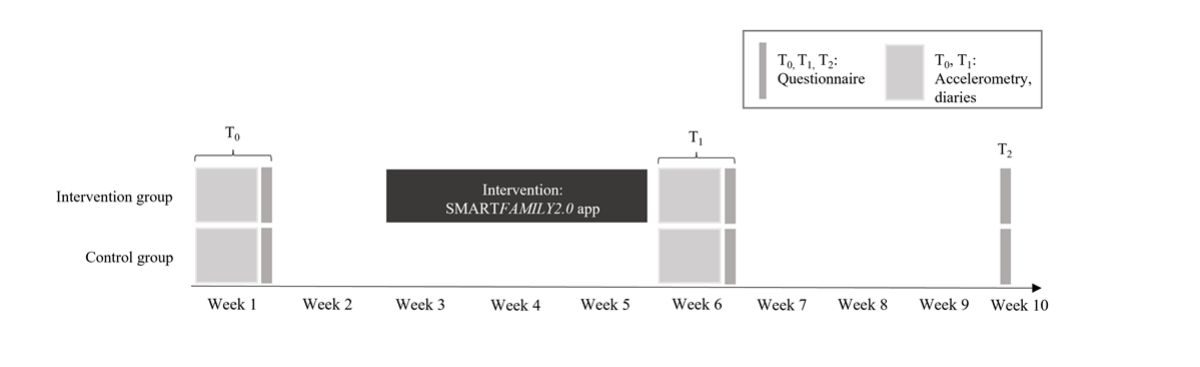
Detailed study design.

The study was conducted and described according to the corresponding study protocol and the CONSORT-EHEALTH (Consolidated Standards of Reporting Trials of Electronic and Mobile Health Applications and Online Telehealth) checklist [77] (Multimedia Appendix 1). A graphical representation of the SF2.0 trial is depicted in Figure 1. Outcome evaluations were conducted at 3 time points: baseline (T_0_), immediately after the 3-week intervention (or absence of intervention) phase (post; T_1_), and 4 weeks after the postmeasurement (follow-up; T_2_). Participants were cluster-randomized into 1 of 2 groups: an intervention group (IG) or a waiting-control group (CG). Given that the study protocol is freely accessible, the study design and measurements will be described very briefly. For a comprehensive overview including screenshots of the app, please consult the study protocol [76].

All eligible members of each family attended an initial visit to the research facility, during which they were provided with detailed instructions on how to use various tools that would be used throughout the study. These tools encompassed accelerometers, which recorded levels of PA, as well as paper diaries for monitoring behavior over time. This procedure was changed to online instructions and mailing of the material during the study due to COVID-19 restrictions. At the end of this initial phase, participants also responded to inquiries about their habits and behaviors during the preceding week, serving as a baseline measurement. This information was subsequently shared with intervention participants to enable them to establish family goals based on this starting point.

Participants assigned to the IG were given specially designed smartphones embedded with our “SF2.0” app. The development of the app followed an iterative approach, incorporating feedback from the prior app, the target audience, and domain experts. Insights from previous research conducted as part of the SMARTACT project and behavioral theories also informed the app’s creation. The programming of the apps was undertaken by the Human-Computer Interaction Workgroup at the University of Konstanz, as a component of the SMARTACT project. To ensure equitable access to technology, study personnel provided all participants with study smartphones. Families were thoroughly briefed on the app’s functionality, with a study manual distributed to aid their comprehension, and study staff were available to address any queries or concerns that arose. Moreover, the accelerometers worn by participants were wirelessly synchronized with the smartphones via Bluetooth low-energy connections.

During the onset of the 3-week intervention, families in the IG were instructed to establish collective weekly goals related to PA and HE. These objectives encompassed achieving a specific step count, engaging in appropriate levels of moderate-to-vigorous PA, planning enjoyable family activities, sufficient FVI, and having joint family meals. Families were instructed to collaborate in formulating these goals based on their previous performance as assessed during the baseline evaluations. To facilitate this process, the goals for the entire family were set on a single smartphone, and the app sent notifications every Sunday to prompt the setting of new goals. During the initial explanation by study staff, participants were encouraged to strive for progressively more ambitious yet attainable goals based on their past behaviors. As part of the program, participants regularly reviewed and adjusted their goals to ensure that they remained challenging yet manageable, fostering ongoing improvement.

The SF2.0 intervention incorporated several gamification features designed to enhance user engagement and promote sustained behavior change. Gamification features included an animated interactive coach that was tailored to participants’ sex and feedback on goal achievement. Components included a point and badge system for completing health-related tasks, personalized progress feedback, and social comparison elements such as leaderboards and peer support interactions. Within the point and badge system, each progress of 10% toward the family goal gained participants 1 star. The app-based intervention also incorporated a JITAI [65,72], delivered by the coach, which prompted users to engage in PA after prolonged periods of inactivity. Inactivity was defined as 1 hour with less than 100 steps and less than 2 minutes with >2 metabolic equivalents (METs) during waking hours. Additionally, the coach provided daily progress updates toward the goals, motivational messages, up to 5 informational and entertaining facts per day, and EMAs regarding sleep quality and core affect. Together, these components were intended to foster motivation, self-monitoring, and social reinforcement in line with self-determination theory and established BCTs such as reinforcement, feedback, and social support. Participants in the nonintervention CG did not receive any materials or communication during the intervention period. All smartphones were collected after the conclusion of the intervention period. Consequently, the IG did not have access to the app during the T_1_ measurement or thereafter.

The app included 14 BCTs [55] for the IG and no BCT for the CG. Intervention BCTs include behavioral goal-setting, prompt review of behavioral goals, prompt self-monitoring of behavior, provide feedback on performance, plan social support or social change, prompt identification as role model or position advocate, set graded tasks, provide shaping, prompt rewards contingent on effort or progress toward behavior, provide rewards contingent on successful behavior, identify barriers or solve problems, teach to use prompts or cues, and prompt review of behavioral goals.

After the intervention or control period ended, participants proceeded to undergo 2 additional testing sessions (T_1_ and T_2_). Throughout the entire process, strict adherence to ethical standards was maintained, and measures were implemented to safeguard personal data. The research study used a single-blind design, whereby participants were unaware of their group assignment. Furthermore, prior to implementation, the survey instruments underwent thorough evaluation to ensure user-friendliness and technical reliability.

### Eligibility Criteria

Households consisting of at least 1 adult caregiver and 1 child older than 10 years of age living together were invited to participate. In cases where applicable, additional siblings, including younger siblings, were also welcome to join the project. All participants were required to possess basic proficiency in operating mobile devices, have the physical capability to engage in PA independently, and effectively communicate in German.

### Ethical Considerations

The research study received full ethics approval from both the University of Konstanz and the Karlsruhe Institute of Technology. Prior to the commencement of the study, all participants, including children, parents, or legal guardians, provided informed written consent. Here, participants were wised up to opt out at any time of the study without any consequences. The study adhered to the guidelines outlined in the Helsinki Declaration. Data were fully pseudonymized (ie, participants generated a code to allow for later data harmonization of the different measurement points). Families who completed the study were provided with a US $46.8 online shopping voucher and an activity tracker for every child of the family as compensation.

### Randomization and Blinding

This study used a cluster-randomized controlled design to compare 2 distinct groups: an IG that received the intervention and a CG that did not receive any treatment. Families who provided consent were randomly assigned to either group using a straightforward allocation scheme suitable for cluster trials [78]. While participants in the IG were aware of the mHealth aspects of the study, participants in the CG were informed about their contribution to an epidemiological examination of PA and overall health. To ensure accurate and reliable measurements, all participants wore accelerometers for 1 week twice within a 10-week duration. Additionally, participants completed various questionnaires during this period.

### Participants

Participants were recruited in schools, school holiday programs, music schools, and sports clubs via personal communication, newspapers, and email distribution lists of the Karlsruhe Institute of Technology. Overall, 55 families (28 CG, 105//209; 27 IG, 104/209 participants) were recruited for the study. In total, 3 families (3 KG, n=12) chose to drop out of the study due to personal reasons before T_0_.

### Measurements

#### PA Measures

##### Device-Based Measured PA (Accelerometry)

The study used hip-worn 3-axial accelerometers (Move 3 or Move 4, Movisens GmbH) placed on the right hip to continuously capture PA data. These accelerometers are recognized scientific research instruments, featuring a measurement range of ±16 g, an output rate of 64 Hz, physical dimensions measuring 62.3 mm×38.6 mm×11.5 mm, and weighing 25 g. Custom epoch lengths of 10 seconds were used to summarize the raw data recorded at 64 Hz. The choice of an epoch length of 10 seconds was based on the belief that shorter epoch lengths are more suitable for estimating vigorous PA and assessing PA in children due to their intermittent movement behavior [59,60]. The validity of a previous accelerometer version (Move 2), which uses similar digital signal processing techniques as the Move 3 or Move 4 models, has been established and considered accurate for assessing steps [79] and energy estimation [80,81] in adults. Participants received instructions and demonstrations from a study instructor regarding the proper handling of the accelerometers. They were directed to wear the accelerometers throughout their waking hours and remove them only during activities such as showering, swimming, or certain sports involving bodily contact to minimize the risk of injuries. The outcomes measured using the accelerometer in this study were MVPA (>3.0 MET) and steps for all participants. MET values were calculated based on the activity class determined by acceleration and barometric signals, which informed the estimation model. The model combined movement acceleration, altitude change, and demographic information to estimate MET values [81]. To be included in the analysis, accelerometer data needed to meet certain criteria. This included a minimum wear time of at least 8 hours per day for at least 4 of the 7 days during the measured week. Nonwear time was calculated in 30-second intervals using an algorithm that incorporated accelerometry and temperature signals over a 10-minute window. This algorithm distinguished between wear time, nonwear time, and sleep, as described elsewhere [64]. Valid measurements were determined by calculating the average of MVPA and steps per valid day and multiplying it by 7 to estimate the total minutes per week.

##### Self-Reported PA

At the conclusion of each week of measurement, all participants were requested to complete the German version of the Global Physical Activity Questionnaire [82]. This questionnaire asked participants to retrospectively report their activities during the previous week. To calculate the results for this study, we multiplied the reported number of days engaged in moderate PA (related to work, travel, and recreation) and vigorous PA (related to work and recreation) by the reported duration of each activity per day. We then added the values for moderate and vigorous PA to obtain the total minutes of MVPA per week. Additionally, a PA diary was used for all participants at time points T_0_ and T_1_. However, the results from the diary were not included in this analysis due to noncomparability with other measures [83,84]. Both parents and children were instructed to independently complete their respective questionnaires and diaries.

#### Fruit and Vegetable Intake

The assessment of FVI involved 2 methods. First, a single item in the questionnaire asked participants to report the total amount of fruits and vegetables consumed in the previous week [85]. Second, a detailed description of food consumption during the time points T_0_ and T_1_ weeks was recorded in a diary. This included information such as the time of the meal, the ingredients, portions of FVI, and whether the meal was consumed within the family or alone.

### Secondary Outcomes

#### Demographics

In the T_0_ questionnaire, demographic information of the participants was collected, including sex, age, height, and weight.

#### Health Status

Perceived general health was assessed using a single item [85].

#### Intrinsic Motivation Toward PA

According to the self-determination theory, the assessment of activity-related self-determination was conducted using the Behavioral Regulation in Exercise Questionnaire-2 (BREQ-2) [86]. BREQ-2 assesses the manifestation of the 5 different regulation modes by the self-determination theory, reflected by the subscales of amotivation (4 items), external (4 items), introjected (3 items), identified (4 items), and intrinsic (4 items) regulations. Responses were made on a 4-point Likert scale, ranging from 0=not true, 1=rather not true, 2=rather true, to 3=true. For the purpose of analysis, a Relative Autonomy Index (RAI) was constructed based on the subscales of the questionnaire. The RAI provides an assessment of the extent to which respondents perceive themselves as self-determined. To calculate the index, each subscale score was multiplied by its assigned weighting, and the weighted scores were then summed. A higher, positive RAI score indicates a higher level of relative autonomy, while a lower, negative score suggests a greater level of controlled regulation.

#### Intrinsic Motivation Toward HE

The Regulation of Eating Behavior Scale [87] was used to assess dietary-related intrinsic motivation. The dimension “integrated regulation” was omitted, resulting in a total of 5 subscales, coded from 0 to 3. A sum score was built analogous to the BREQ-2.

#### Intention

Intention toward PA and FVI as well as intention to use an app for PA and HE were assessed using single items [88].

#### Self-Efficacy for PA and HE

To assess activity-related self-efficacy and dietary-related self-efficacy, the study used health-specific self-efficacy scales. These scales consisted of 5 items for each behavior-related dimension [89]. Participants were asked how certain they are to handle different health-specific barriers. Responses were given on a 4-point Likert scale, ranging from 1=very uncertain, 2=uncertain, 3=certain, to 4=very certain. A sum score was built for both scales.

#### Family Health Climate

To assess shared perceptions and cognitions regarding health behaviors, the study used the Family Health Climate (FHC) scale [90]. This scale consists of 2 separate scales: the FHC-PA scale and the FHC-nutrition scale. FHC-PA contains 14 items, which are assigned to the 3 subscales of value (5 items), cohesion (5 items), and information (4 items). FHC-nutrition includes 17 items, comprising the 4 subscales of value (4 items), cohesion (5 items), consensus (3 items), and communication (5 items). Responses for each dimension were scored on a 4-point Likert scale ranging from 0not true, 1=rather not true, 2=rather true to 3=true. Sum scores were built for both scales.

#### Joint PA and Meals Within the Family

Joint PA and nutrition were assessed using a single item that inquired about the number of activities and meals in which the entire family participated during the previous week. The mean value per family was calculated and used for the analysis.

### Statistical Analysis

The analyses were run with different packages of R (R Foundation for Statistical Computing) and RStudio (RStudio, Inc) [73]. The package *ggplot2* was used for visualizations [91], following the instructions of Allan et al [92]. Mixed models were calculated using the package *lmerTest* [93], with participants (level 1) nested in families (level 2) to acknowledge the hierarchical structure of the data. The result tables of the regression analyses were generated using the package *sjPlot* [94]. Here, 7 final models were calculated, 1 with each measurement method and outcome parameter (1 steps, 2 MVPA, 2 FVI intake per week, 1 joint PA, and 1 joint nutrition) as dependent variables. Assumptions were checked using the visualization of the *performance* package [95]. A hierarchical approach was used for the inclusion of the control variables, and the model fit was assessed with the Akaike information criterion for sensitivity analysis.

The predictor group (ie, control=0 and intervention=1)×time (dummy coded with T_0_ as reference for T_1_ and T_2_) was included in the models to evaluate the interaction effect (main effect) of the intervention on the 7 outcome parameters. To assess sensitivity regarding the additional variables, the secondary outcome parameters self-efficacy, intention, intention for app use, intrinsic motivation, and the family health climate were added, either referring to PA or FVI depending on the outcome. Furthermore, the control variables health status, population (adult=0, children=1), sex (0=male, 1=female), and nonwear time per week—only for the device-based measured PA models—were tested for inclusion in the random effect models. Additionally, the inclusion of random slopes and random intercepts was evaluated based on the model fit. The level of statistical significance was set a priori to α<.05

## Results

### Data Availability and Participant Characteristics

**Figure 2:**
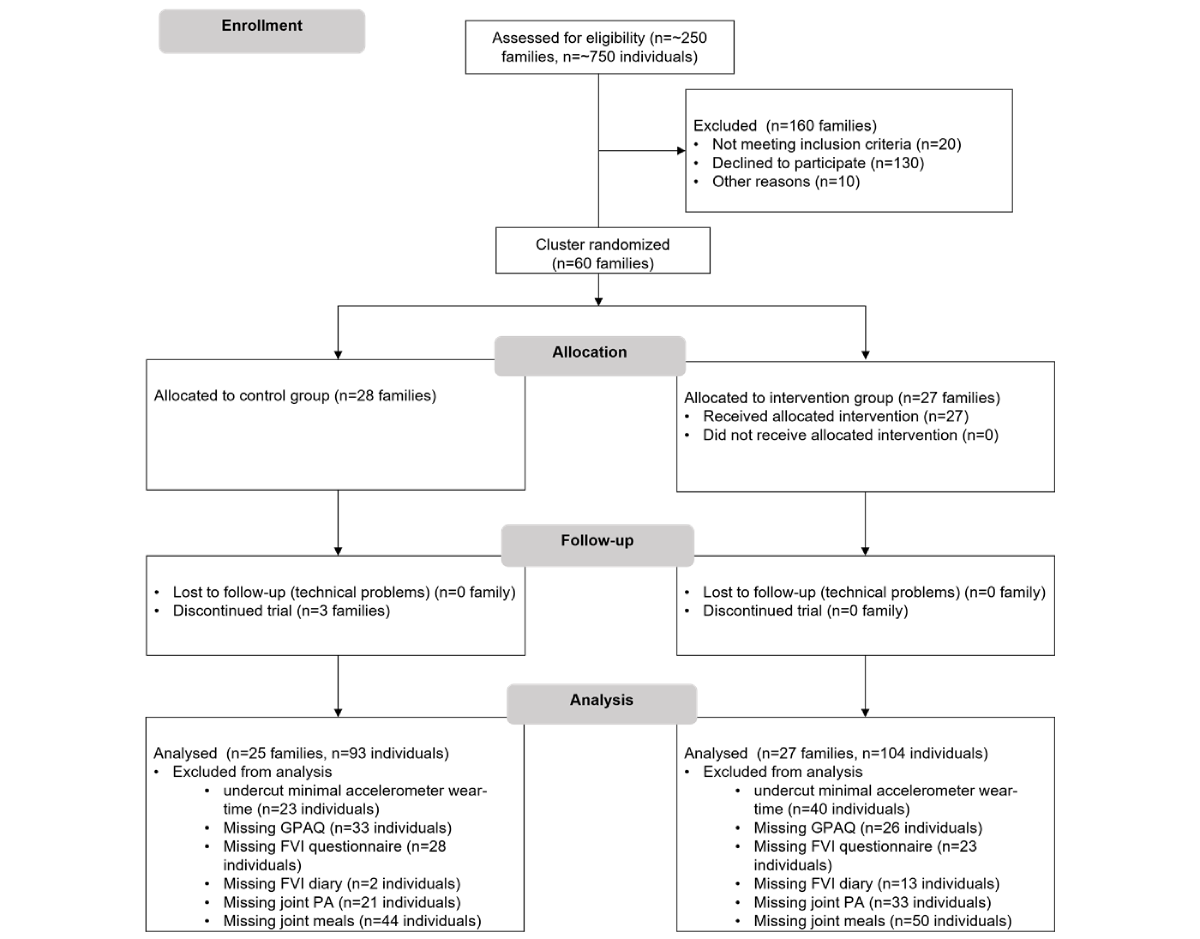
Participant flow diagram. FVI: fruit and vegetable intake; GPAQ: Global Physical Activity Questionnaire; PA: physical activity.

In total, 52 families (female adults: 47/96, 49% and male adults: 49/96, 51%; and female children: 47/101, 47% and male children: 53/101, 53%) participated in the study. Technical issues with the app during the intervention, insufficient wear time of the accelerometer (ie, less than 4 days with more than 8 hours wear time), and missing data for the self-reported items led to the inclusion of a different number of participants for each calculated model (depending on the outcome variables; Multimedia Appendices 2 and 3). For a detailed overview, see the flow diagram in Figure 2.

Participant characteristics of the 52 families separated by group (control vs intervention), population (children vs adults), and sex (male vs female) are displayed in Table 1. Descriptive results for all included outcomes and predictors can be found in Table 2.

All control variables (except nonwear time for MVPA and steps and population for FVI diary) improved the model fit based on Akaike information criterion and were therefore included in the final sensitivity models, with the exception of the joint models, where only population improved the model fit. Random slopes were not supported by the data, but random intercepts were used for all models. Sensitivity analysis showed no difference in patterns for the effectiveness of the intervention (Tables S1-S7 in Multimedia Appendix 3). Therefore, only the main models are reported. Data and code are available at the Open Science Framework [96].

**Table 1:** Participant characteristics of the 52 families included in the *SMARTFAMILY2.0* trial.

	Control group					Intervention group			
	Child		Adult		Child			Adult	
	Male	Female	Male	Female	Male		Female	Male	Female
Values, n (%)	25 (50)	25 (50)	23 (53)	20 (47)	29 (57)		22 (43)	26 (49)	27 (51)
Age (years), mean (SD)	11.5 (3.64)	10.4 (2.58)	45.1 (5.92)	46.4 (6.09)	11.4 (2.73)		12.1 (3.63)	45.0 (5.11)	46.7 (4.98)
BMI (kg/m^2^), mean (SD)	18.9 (3.68)	17.9 (3.19)	24.3 (3.86)	27.2 (4.51)	17.2 (3.12)		18.5 (2.82)	23.5 (3.66)	27.1 (3.66)

**Table 2:** Descriptive results of the 52 families included in the *SMARTFAMILY2.0* trial.

	Control group							Intervention group					
	T_0_		T_1_		T_2_		T_0_			T_1_		T_2_	
	Child, mean (SD)	Adult, mean (SD)	Child, mean (SD)	Adult, mean (SD)	Child, mean (SD)	Adult, mean (SD)	Child, mean (SD)		Adult, mean (SD)	Child, mean (SD)	Adult, mean (SD)	Child, mean (SD)	Adult, mean (SD)
Steps (counts per week)	57000 (26200)	55700 (17800)	64100 (23000)	54600 (17100)	—^a^	—	64600 (25900)		54000 (14500)	61300 (24100)	49800 (20100)	—	—
MVPA^b^ (minutes per week)	579 (346)	621 (236)	729 (351)	631 (221)	—	—	651 (358)		644 (177)	587 (311)	572 (205)	—	—
nwt^c^ (minutes per week)	4990 (538)	4330 (671)	4810 (660)	4380 (596)	—	—	4920 (535)		4400 (561)	4990 (700)	4540 (600)	—	—
GPAQ MVPA^d^ (minutes per week)	988 (1020)	980 (1230)	1200 (1020)	995 (860)	1430 (954)	1410 (1270)	1160 (1380)		725 (687)	1170 (1080)	869 (644)	1050 (742)	990 (614)
FVI diary^e^ (portions per week)	20.7 (14.5)	20.1 (11.6)	18.0 (11.8)	19.7 (10.8)	—	—	21.5 (13.1)		20.5 (10.0)	22.0 (13.3)	24.0 (10.1)	—	—
FVI quest^f^ (portions per week)	19.3 (11.0)	15.8 (11.8)	17.5 (11.2)	17.0 (12.2)	19.2 (12.2)	16.8 (9.96)	20.9 (14.7)		17.8 (11.3)	20.0 (15.1)	20.7 (11.3)	16.3 (8.77)	19.0 (10.1)
Health	4.20 (0.808)	3.70 (0.803)	4.17 (1.11)	3.79 (0.645)	4.33 (0.816)	3.79 (0.592)	4.26 (0.828)		3.91 (0.946)	4.11 (1.04)	4.00 (0.799)	4.41 (0.670)	3.91 (0.686)
M intrinsic PA^g^ (RAI^h^)	35.3 (14.1)	32.8 (18.8)	35.6 (13.0)	35.5 (17.4)	36.2 (14.7)	40.2 (12.9)	34.7 (14.7)		36.4 (12.6)	32.3 (18.4)	34.9 (13.5)	29.3 (20.1)	33.1 (13.1)
M intrinsic NU^i^ (RAI)	9.86 (19.7)	20.0 (17.9)	8.94 (19.1)	2.90 (11.7)	12.5 (20.6)	23.5 (18.9)	11.6 (19.0)		22.0 (15.7)	10.5 (20.7)	5.34 (10.5)	6.61 (22.4)	17.9 (18.0)
int PA^j^	1.94 (1.02)	2.44 (1.22)	1.96 (0.932)	2.10 (1.25)	2.18 (1.07)	1.94 (0.886)	2.08 (0.997)		2.11 (0.934)	1.98 (0.917)	2.04 (0.922)	2.05 (0.973)	2.02 (1.07)
int NU^k^	2.78 (0.840)	2.72 (0.854)	2.66 (0.939)	2.81 (0.890)	2.73 (0.719)	2.74 (0.790)	2.57 (0.855)		2.79 (0.793)	2.37 (0.878)	2.88 (0.761)	2.56 (0.776)	2.89 (0.840)
int app PA^l^	2.68 (1.49)	3.19 (1.20)	2.96 (1.28)	3.00 (1.27)	2.94 (1.46)	3.35 (1.30)	3.10 (1.47)		3.47 (1.26)	3.09 (1.24)	3.04 (1.24)	3.10 (1.43)	2.89 (1.20)
int app NU^m^	2.28 (1.23)	2.72 (1.32)	2.38 (1.24)	2.88 (1.25)	2.55 (1.30)	2.85 (1.28)	2.96 (1.39)		2.94 (1.25)	2.80 (1.31)	2.58 (1.13)	2.61 (1.38)	2.38 (1.05)
FHC^n^ NU^o^	51.6 (7.41)	51.7 (7.01)	50.0 (8.39)	51.0 (7.08)	51.5 (7.46)	51.1 (7.30)	50.6 (8.06)		51.6 (8.82)	48.8 (9.52)	50.0 (8.88)	48.8 (9.00)	51.0 (7.81)
FHC PA^p^	39.0 (7.92)	39.7 (7.63)	38.6 (8.03)	38.7 (7.32)	39.2 (8.37)	40.1 (7.35)	38.2 (7.91)		38.2 (8.33)	38.5 (7.54)	36.9 (7.78)	37.1 (8.34)	37.5 (8.50)
Self-efficacy NU (RAI)	12.0 (3.52)	12.9 (4.23)	12.0 (3.22)	13.0 (3.19)	12.7 (4.13)	12.8 (2.97)	13.4 (3.52)		13.6 (2.84)	12.5 (3.67)	14.2 (2.95)	12.5 (3.46)	13.6 (2.47)
Self-efficacy PA (RAI)	13.6 (3.71)	12.8 (3.53)	13.5 (3.11)	12.7 (3.35)	12.6 (3.53)	13.2 (3.11)	14.6 (2.71)		14.0 (3.04)	13.4 (3.22)	13.0 (3.25)	13.5 (3.18)	13.0 (2.73)
Joint PA	2.00 (3.40)	1.84 (2.85)	3.69 (5.31)	3.03 (4.00)	5.12 (8.24)	3.58 (3.72)	0.960 (1.48)		1.19 (1.78)	3.97 (5.29)	3.08 (4.15)	7.89 (7.82)	3.13 (4.37)
Joint NU	7.52 (4.39)	8.95 (4.26)	9.96 (3.13)	9.54 (2.78)	13.5 (6.00)	14.1 (4.64)	8.20 (5.54)		7.62 (4.67)	9.32 (5.79)	9.83 (5.16)	9.78 (6.82)	12.4 (5.15)

^a^Not available.

^b^MVPA: moderate to vigorous physical activity.

^c^nwt: nonwear time.

^d^GPAQ MVPA: self-reported parameter MVPA measured by the Global Physical Activity Questionnaire.

^e^FVI diary: fruit and vegetable intake measured by diary.

^f^FVI quest: fruit and vegetable intake measured by a single-item questionnaire.

^g^M intrinsic PA: intrinsic motivation toward physical activity.

^h^RAI: Relative Autonomy Index.

^i^M intrinsic NU: intrinsic motivation toward nutrition.

^j^int PA: intention toward changing physical activity.

^k^int NU: intention toward changing nutrition.

^l^int app PA: intention to change physical activity with an app.

^m^int app NU: intention to change nutrition with an app.

^n^FHC: Family Health Climate.

^o^NU: nutrition.

^p^PA: physical activity.

### Effect of the Intervention on PA

**Figure 3:**
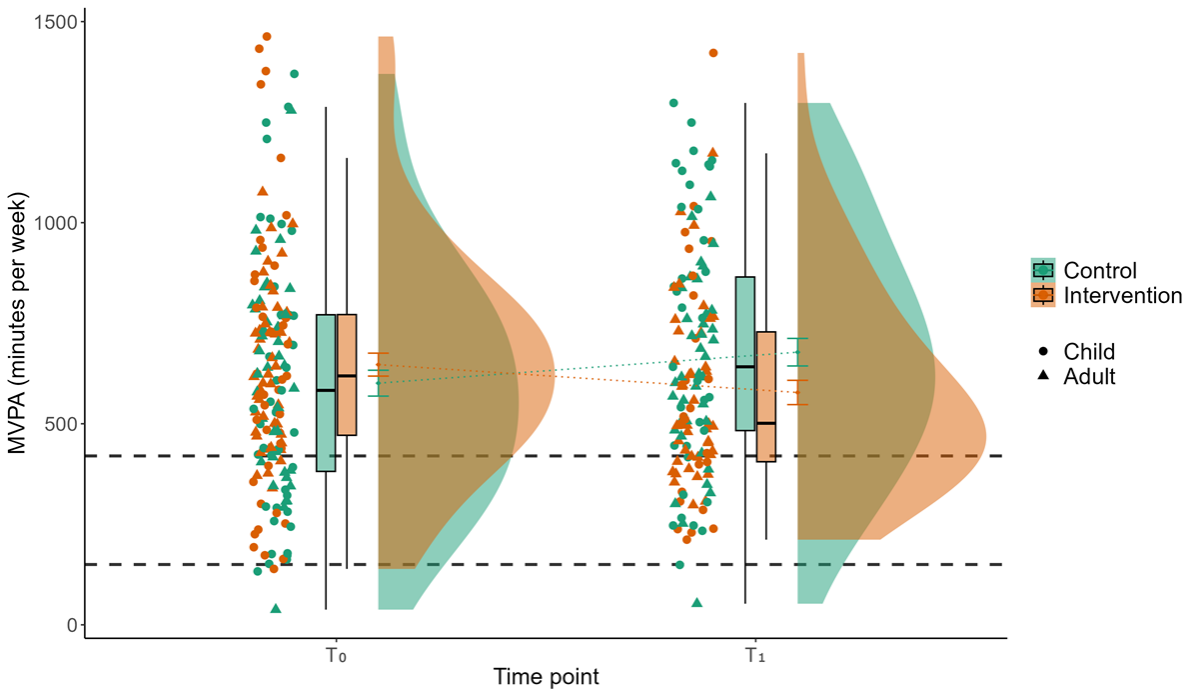
Interaction effect of group×time for device-based measured physical activity for the parameter minutes of MVPA per week. Displayed is the mean MVPA (y-axis) of 180 participants during 1 week of baseline measurement (T_0_) and 1 week of postmeasurement after a 3-week intervention or waiting period (T_1_) for the control group (green) and the intervention group (red), stratified by children and adults. The gray dashed lines represent the physical activity recommendations for children (420 minutes per week) and adults (150 minutes per week). MVPA: moderate to vigorous physical activity.

**Figure 4:**
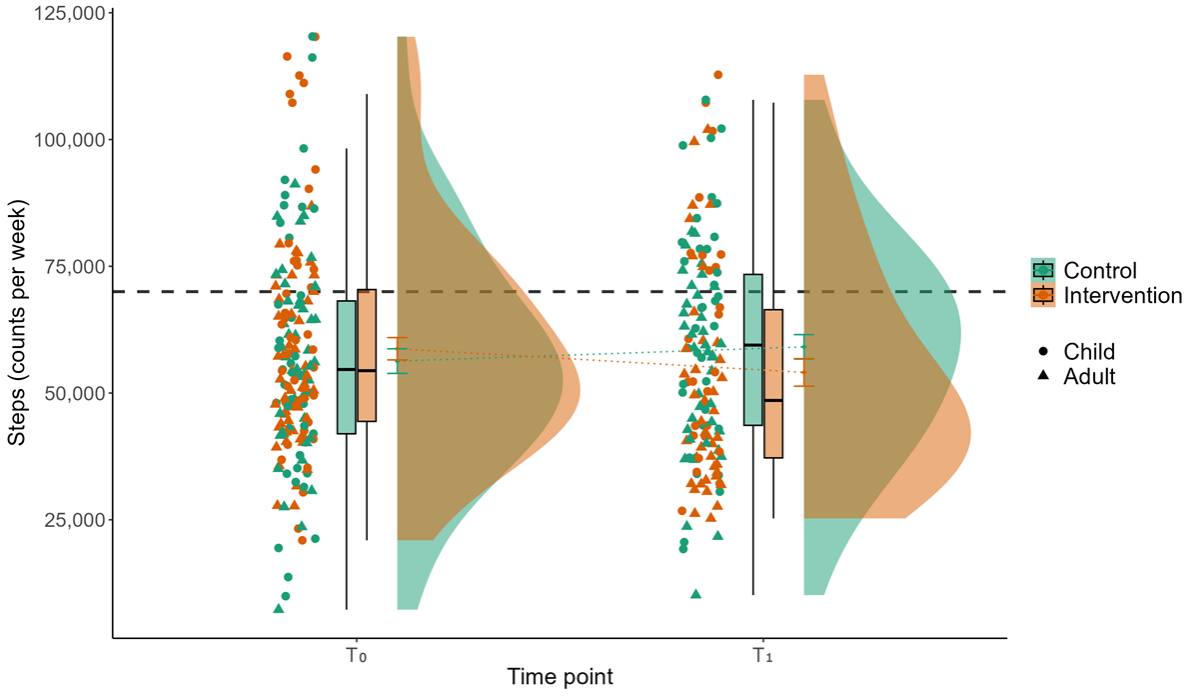
Interaction effect of group×time for device-based measured physical activity for the parameter steps per week (steps). Displayed is the mean step count (y-axis) of 180 participants during 1 week of baseline measurement (T_0_) and 1 week of postmeasurement after a 3-week intervention or waiting period (T_1_) for the control group (green) and the intervention group (red), stratified by children and adults. The gray dashed line represents the commonly used step recommendation of 10,000 steps per day (70,000 steps per week).

Results of the linear mixed models indicate significant main effects for the interaction of group with time in device-based measured MVPA between T_0_ and T_1_ in favor of the CG (*P*=.02; β=–.50). Steps show no significant main effect but the β point in the same direction as for device-based measured MVPA. Self-reported MVPA with the Global Physical Activity Questionnaire reveals no significant interaction of group×time but a significant time effect between T_0_ and T_2_ (*P*=.008; β=.21). For all main results, see Tables S1-S3 in Multimedia Appendix 2. Figures 3 and 4 display the descriptive results for the device-based measured PA outcomes, MVPA and step count. As displayed by the gray dotted lines in Figure 3, both mean and median values are clearly above the recommendation for MVPA [4] for both children (ie, 60 minutes per day on average, 420 minutes per week) and adults (ie, 150 minutes per week) in both groups and at both measurement periods. For steps, mean and median values are below the commonly used 10,000 steps per day (70,000 steps per week) goal [97,98] for all participants (Figures 3 and 4 ).

### Effect of the Intervention on FVI

**Figure 5:**
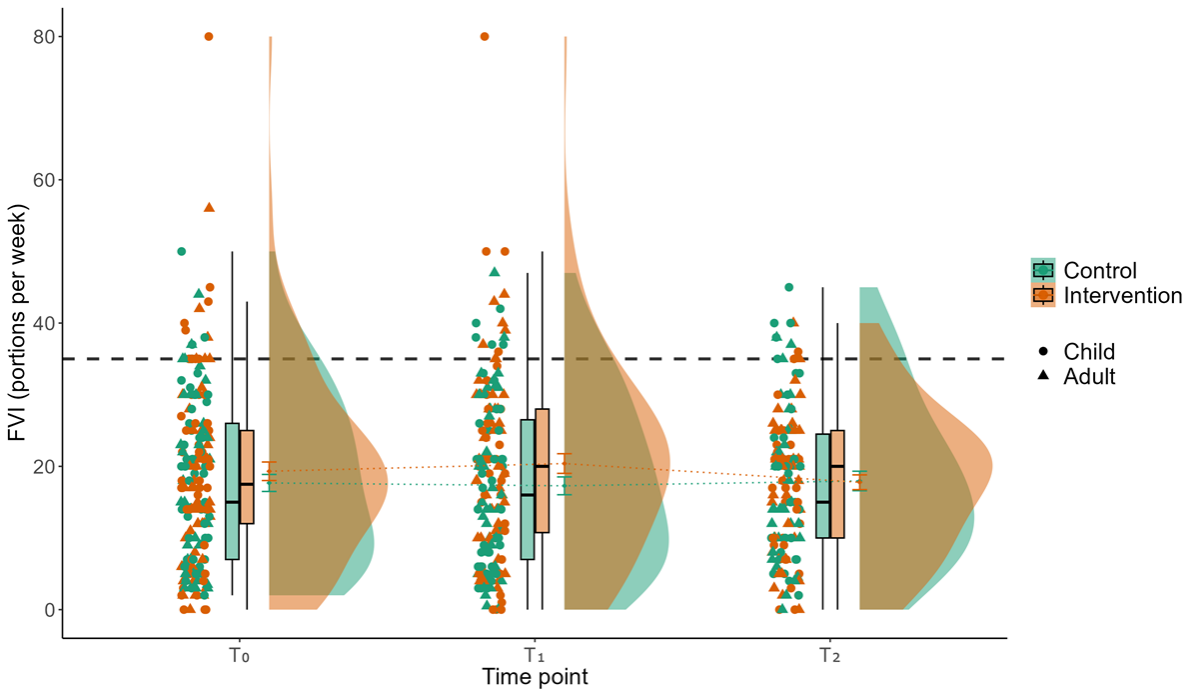
Interaction effect of group×time for the parameter FVI per week assessed by questionnaire. Displayed is the mean FVI (y-axis) of 197 participants related to the week of baseline measurement (T_0_), the week of postmeasurement after a 3-week intervention or waiting period (T_1_), and the week of follow-up measurement (T_2_) for the control group (green) and the intervention group (red), stratified by children and adults. The gray dashed line represents the recommendation for a daily FVI of 5 portions (35 portions per week). FVI: fruit and vegetable intake.

Results of the linear mixed models indicate a significant interaction of group×time, concerning FVI reported by the diary between T_0_ and T_1_ in favor of the IG (*P*=.03; β=–.35). There is no significant main effect for FVI assessed via questionnaire (Tables S4 and S5 in Multimedia Appendix 2). Figure 5 displays the descriptive results for self-reported FVI per week assessed by the questionnaire. Here, both mean and median values are clearly below the recommended FVI of 5 portions per day (35 portions per week) [99].

### Effect of the Intervention on Joint PA and Meals

Results of the linear models indicate a significant effect for group×time for both T_0_ to T_1_ (*P*=.02; β=.18) and T_0_ to T_2_ (*P*=.008; β=.29) in favor of the IG for joint PA. Regarding joint meals, a significant group×time interaction was found for T_0_ to T_1_ in favor of the IG (*P*=.004; β=.14), and a significant group×time interaction was found for T_0_ to T_2_ in favor of the CG (*P*<.001; β=–.34). Additionally, all time effects except for joint meals T_0_ to T_1_ were significant with a β between .21 and 1.20. All results are displayed in Tables S6 and S7 in Multimedia Appendix 2.

## Discussion

### Principal Findings

The SF2.0 trial evaluated the effectiveness of a refined mHealth intervention to increase PA and HE in a family setting. Extending previous research, the behavior of children and parents was targeted to induce individual behavior changes that are anchored in daily family life. Moreover, besides a theoretical foundation, several BCTs were additionally included, which contribute to the fulfillment of basic psychological needs according to the self-determination theory [100]. Overall, there was no significant intervention effect of our app for PA, independent of the measurement method, but for FVI as measured by the diary. However, participants were not able to maintain this effect 4 weeks after intervention cessation. The explorative results for joint PA point to the potential effectiveness of the collaborative mHealth intervention.

Compared with previous mHealth interventions targeting PA and HE, SF2.0 incorporates a broader range of evidence-based BCTs, including self-monitoring, tailored feedback, social support, and gamification. Earlier interventions have typically focused on either PA or dietary behavior alone, and many relied on static content or 1-way messaging.

For example, a systematic review of text message interventions for PA found that many studies used 1-way motivational or educational messages, with some interventions relying solely on text messages as the primary delivery tool, often without interactive or tailored features [101]. Similarly, a systematic review of digital interventions for adolescents reported that most interventions were web-based and primarily focused on either PA or diet, with limited use of interactive or dynamic content. Text message and email interventions were few and generally less effective at changing behaviors [102]. Reviews of technology-supported interventions for adults and survivors of cancer also highlight that many early interventions delivered static web modules or print materials, with limited facilitation or interactivity [103].

In contrast, SF2.0 uses adaptive personalization and interactive features designed to enhance user motivation and sustained engagement. These additions reflect an evolution from information delivery toward dynamic, theory-driven behavior change strategies. Furthermore, while prior studies have often reported short-term improvements with declining engagement over time, SF2.0’s design aims to maintain user interaction through its self-determination–based gamified framework, potentially supporting longer-term behavioral adherence.

### PA and HE

Regarding PA results, it is noteworthy that an interaction effect occurred for device-based measured MVPA in favor of the CG, and the same tendency was found in the steps measure. At first sight, this seems arbitrary. However, a closer look at the data reveals that the sample was already highly active at baseline, however, coincidentally just as low regarding their FVI. Regarding guideline adherence, participants exceeded the recommended amount of 420 minutes of MVPA per week by 180 minutes on average. However, they simultaneously deceed the recommended amount of 35 portions of fruits and vegetables by 15 (diary) or 17.5 (questionnaire) portions. While our sample can be classified as being highly active in an international comparison [94,104], they cannot be classified as equally healthy regarding their eating behavior. In a German-wide representative sample, 17% of 6- to 11-year-olds and 20% of 12- to 17-year-olds reached the recommendation on fruit (vegetable) consumption [14,105]. Our intervention was able to evoke a positive intervention effect on FVI based on diaries, as there was enough room for improvement in contrast to PA levels. Surprisingly, this effect was not present in FVI assessed via questionnaires. This elucidates the need to interpret findings with caution regarding measurement accuracy and construct validity. Research shows that the same construct measured with different methods is likely to lead to different results [106,107], and a comparison within the current data shows a limited comparability between device-based and self-reported measures [83,84]. Additionally, a secondary data analysis on the JITAI within the IG of this study points to promising results concerning PA enhancement in the subsequent hour after a prolonged period of inactivity [74]. This highlights the importance of considering both the time frame and the specific aim of the intervention. Improvements that are evident in hour-to-hour analyses—which can be crucial for detecting health benefits [108]—may not be observable when only pre- to posttest changes at the weekly level are examined. Moreover, the different findings regarding PA and FVI support the notion that both health behaviors must be viewed and targeted independently in interventions, as they do not seem to be related.

In contrast to MVPA and step findings, joint PA within the family revealed a positive intervention effect, that is, our app supported the families to be active together. These results show that it is of importance to interpret the quality of PA in addition to the amount and intensity. Studies have found the positive influence of social interaction for habit formation [109,110]. Hence, it is of high importance to facilitate family events of PA to teach children and adolescents the fun and enjoyment of PA in social groups. For joint meals, however, the effect of T_0_ to T_1_ was in favor of the IG and T_0_ to T_2_ was in favor of the CG, but an increase over time was observable in all groups.

Taken together, the hypothesis that HE would increase due to the intervention was supported by diary data. For PA, no or even a partially reversed intervention effect could be detected. However, this is in line with previous digital health studies also revealing heterogeneous results, with a majority of studies finding at least some significant benefit of interventions [50,53]. However, it needs to be noted that the current sample was exceedingly high in their amount of PA, but coincidentally just as low regarding their FVI, which might have additionally influenced the results.

### Family-Based Interventions

Research on mHealth interventions for families is scarce, particularly those involving randomized controlled trials. Existing studies often focus on digital interventions between parents and children, but the results are mixed and sometimes combined with nondigital approaches. These studies tend to emphasize children’s behavior rather than considering the entire family [111,112]. A recent review highlighted the effectiveness of digital interventions in preventing childhood obesity, with only 2 of the included studies specifically addressing mHealth interventions [113]. In one study, an app for self-monitoring weight and goal setting led to greater weight reduction compared to standard care after 6 months in children with obesity [114]. Another study aimed to enhance fundamental movement skills in 3- to 6-year-old children and observed improvements after a 2-month intervention period [115]. However, not all studies reported positive effects. Of the 7 studies included in another review [116], 2 pilot studies reported significant improvements in PA, 3 studies found evidence for some improvements in PA measures, for example, collaborative PA of children and parents, and the remaining 2 studies found no evidence for an effect. Interestingly, 1 study found that adolescent dropout rates were more than 12 times higher when parents stopped using the app. In this regard, analyzing family behavior and dyadic relationships will be a promising approach for future investigations. Studies suggest that family meal practices and values can support HE [101,117] and that the frequency of shared family meals is significantly related to nutritional health in children and adolescents [31]. The current results do not support the assumption that joint PA or joint meals (ie, some kind of “quality time” within the family) impacts quantitative PA or HE behavior. However, it needs to be acknowledged that joint PA was rather low in our sample (only about 1 joint activity per week), and the enjoyment of PA or attitude toward PA was not an outcome variable in the current trial.

### Strengths and Limitations

The main strengths of the SF2.0 intervention are that (1) it collaboratively targets the family, (2) it is designed as a cluster-randomized controlled trial, (3) it is theory-based, and (4) it incorporates 14 different BCTs, 4 more than the prior version of the app. Further, (5) the goal setting in the app is ad libitum selected by the family to fit their schedule and preferences with guidance from the results of the first measurement week. This empowers the families to set self-selected goals, which have been found to increase motivation and adherence [118,119]. Moreover, in this recent app version, (6) JITAIs were included to change behavior when participants were most prone to it [65,72,74]. Additionally, (7) gamification features were expanded through an avatarized coach, which asked questions for (8) EMA, provided support with goal-setting challenges, and delivered health literacy information. The gamification components of SF2.0 may have contributed to the intervention’s effectiveness by promoting intrinsic motivation and engagement. Features such as progress tracking, achievement badges, and social comparison likely enhanced users’ sense of competence and relatedness, which are key drivers of sustained behavior change. By making health-promoting behaviors more interactive and rewarding, gamification may have facilitated habit formation and long-term adherence to PA and dietary goals. Another strength regarding the evaluation is (9) that multiple outcome measures of self-reported and device-based measured PA were considered. This is important, as these measures are known to yield different results, and including multiple measurement tools improves the plausibility of the results [83,120]. Finally, using (10) advanced statistical methods to consider the nested structure of the data by applying multilevel analyses enhanced the accuracy of the results, as it considered the variance based on the clustering of participants in families. The intraclass coefficients highlight the importance of the family for the outcome parameters, as the data were correlated between 0.16 and 0.39 within the families for the individual outcomes.

Some limitations have to be acknowledged. Regarding our sample, family sizes and ages within families have been very diverse, while the required sample size by the a priori power analysis of 52 families and 156 participants has been met [76]. However, there is a lack of knowledge about how these composite family structures interfere with results regarding behavior change or the accomplishment of healthy lifestyles. For example, it might be assumed that older parents might be more aware of healthy food choices, as they consider healthy nutrition as being more important for themselves than their younger counterparts [121]. Advanced paternal age is associated with increased risk, and younger paternal age with decreased risk of children’s eating disorders [122]. A further restriction might be the age range, especially for children and adolescents. Since this study includes the whole family, children of different ages and with different needs and perceptions were addressed similarly by the app, which might have affected intervention effects. Future interventions should aim to address the individual needs of the family members and tailor the intervention more specifically to the participants. Another important factor concerning the current sample, which could explain the lack of significant intervention effects for PA, was probably the highly active sample with around 600 minutes of MVPA per week and person, which already fulfilled the guidelines of the World Health Organization for PA [4]. This is in contrast to recent research about PA guideline fulfillment, which reliably reveals only small portions of participants fulfilling the PA recommendations, with lower amounts with increasing ages [9-11]. This skewness of our sample was also apparent regarding body composition and HE behavior [123]. With BMIs between 17 and 19 kg/m^2^ in children, and between 23 and 27 in adults, our sample was of normal weight. Studies have shown that intervention effects can be expected to be higher in people with overweight or obesity, both for HE and PA interventions [124]. This might be a probable explanation for the absence of intervention effects (ie, there was no or only little room for improvement in our sample).

An additional aspect was that the participants had to use the provided smartphones instead of their own for equality and data security reasons, which can be burdensome. However, previous research showed no differences in engagement between participants with their own smartphones versus additional smartphones [125]. If a program aims for long-term sustainability beyond a scientific scope, the use of an additional phone must be considered very carefully.

Another potentially limiting factor is the comparably short duration of the intervention. Based on literature regarding behavior change theories (ie, the transtheoretical model) [126], an intervention duration of 3 weeks might not have been sufficient [53]. However, mHealth intervention studies even revealed significant behavior change effects with intervention durations of only 1 [127], 2 [128], and 3 weeks [129]. In a similar vein, a recent meta-analysis on mobile apps for diet showed that interventions with longer duration were not generally more effective [130].

Moreover, as we examined families in their natural setting, there are also practical constraints. In Germany, a continuous school period lasts a maximum of 6 to 8 weeks, followed by a vacation period. To conduct the core assessments including pre- and posttesting accelerometry (please see also Figure 1) during 1 continuous school period, an intervention period longer than 3 weeks was not feasible for the study design. Longer intervention periods would inevitably mean that there is a cofounding between the assessment period (school time vs vacation). Furthermore, data were gathered during the COVID-19 pandemic, but we did not test any participants in a lockdown situation, that is, where they were not allowed to leave the house. However, homeschooling and home office might have influenced the results, both in children and adults. Especially in Germany, research showed that COVID-19 increased the amount of PA in children and adolescents [120], depending on population density [131]. Independent of age, sex, and country, however, a declining trend in PA was found [132].

### Conclusions

Taken together, the evaluation of the SF2.0 trial expands the existing body of evidence, as it investigated the influence of a theory-based mHealth intervention targeting PA and HE in a collective family-based setting. Yet, no evidence for the effectiveness of the trial has been found for PA, but diary data of HE and joint PA showed improvements due to app use. The finding regarding PA might be attributable to an initially active and lean sample. Future evaluations of interventions should therefore also consider (1) methods that go beyond pre- and post–follow-up designs to account for the timeliness and complexity of mHealth interventions, (2) recruiting participants of all activity and weight levels, and (3) control for or restrict ages of children and parents.

## Appendix

Multimedia Appendix 1 CONSORT-eHEALTH (V1.6.1) checklist.

Multimedia Appendix 2 Results of the main analyses.

Multimedia Appendix 3 Results of the sensitivity analyses.

## Abbreviations

BCT: behavior change technique
BREQ-2: Behavioral Regulation in Exercise Questionnaire-2
CG: control group
CONSORT-EHEALTH: Consolidated Standards of Reporting Trials of Electronic and Mobile Health Applications and Online Telehealth
EMA: ecological momentary assessment
FHC: Family Health Climate
FVI: fruit and vegetable intake
HE: healthy eating
IG: intervention group
JITAI: just-in-time adaptive intervention
MET: metabolic equivalent
mHealth: mobile health
MVPA: moderate to vigorous physical activity
PA: physical activity
RAI: Relative Autonomy Index
SF: *SMARTFAMILY*

## Data Availability

The datasets generated or analyzed during this study are available in the Open Science Framework repository [96].
