# Supplementary material for: A Refined Mobile Health Intervention (SMARTFAMILY2.0) to Promote Physical Activity and Healthy Eating in a Family Setting: Randomized Controlled Trial
Source: JMIR Mhealth Uhealth. 2025 Dec 15;13:e65558. doi: 10.2196/65558 (PMC12750077; doi:10.2196/65558)
Supplement: Multimedia Appendix 2 [file mhealth_v13i1e65558_app2.docx]

**Table S1.** Multilevel model analysis for the influence of the three week intervention period on self-reported physical activity Global Physical Activity Questionnaire) in adults and children. Displayed are the results of the group (control = 0, intervention = 1) x time (dummy coded with T_0_ as reference for T_1_ and T_2_) interaction. All results are displayed using the raw estimates (minutes of moderate to vigorous physical activity (MVPA) per week), the standardized Beta (β), 95% confidence intervals (CI), and standardized (std.) 95% CI. Additionally, the within-person variance (σ^2^), the between-person variance (τ_00_ _family_), the intraclass correlation coefficient (ICC), the number of families (N _family_), the number of observations, and the marginal and conditional R² are displayed.

|  | **Global Physical Activity Questionnaire**  **MVPA/week** | | | | |
| --- | --- | --- | --- | --- | --- |
| *Predictors* | *Estimates* | *β* | *CI* | *std. CI* | *p* |
| (Intercept) | 964.32 | -0.11 | 701.49 – 1227.15 | -0.29 – 0.08 | **<0.001** |
| group | -14.20 | -0.01 | -376.17 – 347.77 | -0.19 – 0.17 | 0.939 |
| timepoint [T1] | 106.84 | 0.09 | -150.40 – 364.08 | -0.09 – 0.27 | 0.415 |
| timepoint [T2] | 383.37 | 0.21 | 98.39 – 668.36 | 0.02 – 0.40 | **0.008** |
| group × timepoint [T1] | -26.65 | -0.01 | -381.64 – 328.34 | -0.19 – 0.17 | 0.883 |
| group × timepoint [T2] | -326.39 | -0.16 | -706.21 – 53.42 | -0.36 – 0.03 | 0.092 |
| **Random Effects** | | | | | |
| σ^2^ | 738733.12 | | | | |
| τ_00_ _family_ | 234954.35 | | | | |
| ICC | 0.24 | | | | |
| N _family_ | 52 | | | | |
| Observations | 516 | | | | |
| Marginal R^2^ / Conditional R^2^ | 0.015 / 0.252 | | | | |

**Table S2.** Multilevel model analysis for the influence of the three week intervention period on device-based measured physical activity (accelerometry using 10 second epochs) in adults and children. Displayed are the results of the group (control = 0, intervention = 1) x time (dummy coded with T_0_ as reference for T_1_) interaction. All results are displayed using the raw estimates (minutes of moderate to vigorous physical activity (MVPA) per week), the standardized Beta (β), 95% confidence intervals (CI), and standardized (std.) 95% CI. Additionally, the within-person variance (σ^2^), the between-person variance (τ_00_ _family_), the intraclass correlation coefficient (ICC), the number of families (N _family_), the number of observations, and the marginal and conditional R² are displayed.

|  | **Accelerometry**  **MVPA/week** | | | | |
| --- | --- | --- | --- | --- | --- |
| *Predictors* | *Estimates* | *β* | *CI* | *std. CI* | *p* |
| (Intercept) | 602.18 | -0.09 | 531.16 – 673.19 | -0.34 – 0.16 | <0.001 |
| group | 40.12 | 0.14 | -58.62 – 138.86 | -0.21 – 0.50 | 0.425 |
| timepoint [T1] | 69.89 | 0.25 | -10.62 – 150.39 | -0.04 – 0.54 | 0.089 |
| group [intervention] × timepoint [T1] | -132.20 | -0.47 | -246.84 – -17.57 | -0.88 – -0.06 | 0.024 |
| **Random Effects** | | | | | |
| σ^2^ | 64353.55 | | | | |
| τ_00_ _family_ | 12609.71 | | | | |
| ICC | 0.16 | | | | |
| N _family_ | 52 | | | | |
| Observations | 314 | | | | |
| Marginal R^2^ / Conditional R^2^ | 0.015 / 0.176 | | | | |

**Table S3.** Multilevel model analysis for the influence of the three week intervention period on device-based measured physical activity (accelerometry using 10 second epochs) in adults and children. Displayed are the results of the group (control = 0, intervention = 1) x time (dummy coded with T_0_ as reference for T_1_) interaction. All results are displayed using the raw estimates (step count per week), the standardized Beta (β), 95% confidence intervals (CI), and standardized (std.) 95% CI. Additionally, the within-person variance (σ^2^), the between-person variance (τ_00_ _family_), the intraclass correlation coefficient (ICC), the number of families (N _family_), the number of observations, and the marginal and conditional R² are displayed.

|  | **Accelerometry**  **step count/week** | | | | |
| --- | --- | --- | --- | --- | --- |
| *Predictors* | *Estimates* | *β* | *CI* | *std. CI* | *p* |
| (Intercept) | 56024.48 | -0.05 | 50258.52 – 61790.43 | -0.32 – 0.21 | **<0.001** |
| group | 2171.88 | 0.10 | -5852.23 – 10195.99 | -0.27 – 0.48 | 0.595 |
| timepoint [T1] | 2425.84 | 0.11 | -3496.38 – 8348.07 | -0.16 – 0.39 | 0.421 |
| group [intervention] × timepoint [T1] | -6750.05 | -0.31 | -15193.01 – 1692.91 | -0.71 – 0.08 | 0.117 |
| **Random Effects** | | | | | |
| σ^2^ | 347374573.42 | | | | |
| τ_00_ _family_ | 106254621.75 | | | | |
| ICC | 0.23 | | | | |
| N _family_ | 52 | | | | |
| Observations | 314 | | | | |
| Marginal R^2^ / Conditional R^2^ | 0.007 / 0.240 | | | | |

**Table S4.** Multilevel model analysis for the influence of the three week intervention period on self-reported fruit and vegetable intake (single item questionnaire) in adults and children. Displayed are the results of the group (control = 0, intervention = 1) x time (dummy coded with T_0_ as reference for T_1_ and T_2_) interaction. All results are displayed using the raw estimates (fruit and vegetable portions per week), the standardized Beta (β), 95% confidence intervals (CI), and standardized (std.) 95% CI. Additionally, the within-person variance (σ^2^), the between-person variance (τ_00_ _family_), the intraclass correlation coefficient (ICC), the number of families (N _family_), the number of observations, and the marginal and conditional R² are displayed.

|  | **Questionnaire**  **fruit and vegetable intake/week** | | | | |
| --- | --- | --- | --- | --- | --- |
| *Predictors* | *Estimates* | *β* | *CI* | *std. CI* | *p* |
| (Intercept) | 17.45 | -0.02 | 14.11 – 20.78 | -0.21 – 0.18 | **<0.001** |
| group | 1.54 | 0.07 | -3.08 – 6.17 | -0.13 – 0.26 | 0.512 |
| timepoint [T1] | -0.23 | 0.06 | -3.05 – 2.59 | -0.11 – 0.23 | 0.873 |
| timepoint [T2] | 0.45 | -0.03 | -2.65 – 3.54 | -0.20 – 0.15 | 0.777 |
| group × timepoint [T1] | 1.75 | 0.07 | -2.18 – 5.68 | -0.09 – 0.24 | 0.381 |
| group × timepoint [T2] | -1.45 | -0.06 | -5.62 – 2.72 | -0.24 – 0.12 | 0.495 |
| **Random Effects** | | | | | |
| σ^2^ | 91.93 | | | | |
| τ_00_ _family_ | 46.78 | | | | |
| ICC | 0.34 | | | | |
| N _family_ | 52 | | | | |
| Observations | 527 | | | | |
| Marginal R^2^ / Conditional R^2^ | 0.009 / 0.343 | | | | |

**Table S5.** Multilevel model analysis for the influence of the three week intervention period on self-reported fruit and vegetable intake (diary) in adults and children. Displayed are the results of the group (control = 0, intervention = 1) x time (dummy coded with T_0_ as reference for T_1_) interaction. All results are displayed using the raw estimates (fruit and vegetable portions per week), the standardized Beta (β), 95% confidence intervals (CI), and standardized (std.) 95% CI. Additionally, the within-person variance (σ^2^), the between-person variance (τ_00_ _family_), the intraclass correlation coefficient (ICC), the number of families (N _family_), the number of observations, and the marginal and conditional R² are displayed.

|  | **Diary**  **fruit and vegetable intake/week** | | | | |
| --- | --- | --- | --- | --- | --- |
| *Predictors* | *Estimates* | *β* | *CI* | *std. CI* | *p* |
| (Intercept) | 20.32 | -0.04 | 16.80 – 23.83 | -0.34 – 0.25 | **<0.001** |
| group | 0.42 | 0.03 | -4.44 – 5.28 | -0.37 – 0.44 | 0.866 |
| timepoint [T1] | -1.59 | -0.13 | -4.29 – 1.10 | -0.36 – 0.09 | 0.246 |
| group x timepoint [T1] | 4.19 | 0.35 | 0.40 – 7.99 | 0.03 – 0.67 | **0.031** |
| **Random Effects** | | | | | |
| σ^2^ | 85.45 | | | | |
| τ_00_ _family_ | 55.76 | | | | |
| ICC | 0.39 | | | | |
| N _family_ | 52 | | | | |
| Observations | 376 | | | | |
| Marginal R^2^ / Conditional R^2^ | 0.018 / 0.406 | | | | |

**Table S6.** Multilevel model analysis for the influence of the three week intervention period on self-reported joint physical activities per week in the families. Displayed are the results of the group (control = 0, intervention = 1) x time (dummy coded with T_0_ as reference for T_1_ and T_2_) interaction. All results are displayed using the raw estimates (number of joint physical activities per week), the standardized Beta (β), 95% confidence intervals (CI), and standardized (std.) 95% CI. Additionally, the within-person variance (σ^2^), the between-person variance (τ_00_ _family_), the intraclass correlation coefficient (ICC), the number of families (N _family_), the number of observations, and the marginal and conditional R² are displayed.

|  | **Questionnaire**  **number of joint physical activities/week** | | | | |
| --- | --- | --- | --- | --- | --- |
| *Predictors* | *Estimates* | *std. Beta* | *CI* | *standardized CI* | *p* |
| (Intercept) | 2.04 | -0.33 | 1.00 – 3.09 | -0.53 – -0.13 | **<0.001** |
| group | -1.08 | -0.15 | -2.52 – 0.36 | -0.35 – 0.05 | 0.142 |
| timepoint [T1] | 0.91 | 0.44 | 0.12 – 1.71 | 0.28 – 0.60 | **0.025** |
| timepoint [T2] | 3.29 | 1.20 | 2.18 – 4.40 | 0.99 – 1.41 | **<0.001** |
| group × timepoint [T1] | 1.31 | 0.18 | 0.15 – 2.48 | 0.02 – 0.34 | **0.026** |
| group × timepoint [T2] | 2.07 | 0.29 | 0.53 – 3.61 | 0.07 – 0.50 | **0.008** |
| **Random Effects** | | | | | |
| σ^2^ | 5.74 | | | | |
| τ_00_ _family_ | 5.14 | | | | |
| ICC | 0.47 | | | | |
| N _family_ | 50 | | | | |
| Observations | 379 | | | | |
| Marginal R^2^ / Conditional R^2^ | 0.196 / 0.575 | | | | |

**Table S7.** Multilevel model analysis for the influence of the three week intervention period on self-reported joint meals per week in the families. Displayed are the results of the group (control = 0, intervention = 1) x time (dummy coded with T_0_ as reference for T_1_ and T_2_) interaction. All results are displayed using the raw estimates (number of joint meals per week), the standardized Beta (β), 95% confidence intervals (CI), and standardized (std.) 95% CI. Additionally, the within-person variance (σ^2^), the between-person variance (τ_00_ _family_), the intraclass correlation coefficient (ICC), the number of families (N _family_), the number of observations, and the marginal and conditional R² are displayed.

|  | **Questionnaire**  **joint meals/week** | | | | |
| --- | --- | --- | --- | --- | --- |
| *Predictors* | *Estimates* | *β* | *CI* | *std. CI* | *p* |
| (Intercept) | 8.47 | -0.18 | 6.92 – 10.03 | -0.42 – 0.06 | **<0.001** |
| group | -0.28 | -0.03 | -2.43 – 1.87 | -0.27 – 0.21 | 0.797 |
| timepoint [T1] | 0.33 | 0.21 | -0.27 – 0.92 | 0.12 – 0.30 | 0.279 |
| timepoint [T2] | 4.70 | 0.69 | 3.89 – 5.51 | 0.56 – 0.83 | **<0.001** |
| group × timepoint [T1] | 1.22 | 0.14 | 0.40 – 2.04 | 0.04 – 0.23 | **0.004** |
| group × timepoint [T2] | -3.07 | -0.34 | -4.26 – -1.88 | -0.47 – -0.21 | **<0.001** |
| **Random Effects** | | | | | |
| σ^2^ | 2.00 | | | | |
| τ_00_ _family_ | 14.31 | | | | |
| ICC | 0.88 | | | | |
| N _family_ | 50 | | | | |
| Observations | 319 | | | | |
| Marginal R^2^ / Conditional R^2^ | 0.090 / 0.888 | | | | |
