# Supplementary material for: A Refined Mobile Health Intervention (SMARTFAMILY2.0) to Promote Physical Activity and Healthy Eating in a Family Setting: Randomized Controlled Trial
Source: JMIR Mhealth Uhealth. 2025 Dec 15;13:e65558. doi: 10.2196/65558 (PMC12750077; doi:10.2196/65558)
Supplement: Multimedia Appendix 3 [file mhealth_v13i1e65558_app3.docx]

**Table S1.** Linear mixed model analysis for the influence of the three week intervention period on self-reported physical activity (Global Physical Activity Questionnaire) in adults. Displayed are the results of the group (control = 0, intervention = 1) x time (dummy coded with T_0_ as reference for T_1_ and T_2_) interaction, and the secondary outcomes self-efficacy, intrinsic motivation, the family health climate, intention for behavior change, intention towards app use for behavior change, (all relating to physical activity), and health status as control variables. Additionally, sex (0 = male, 1 = female) is included as a control variable. All results are displayed using the raw estimates (minutes of moderate to vigorous physical activity (MVPA) per week), the standardized Beta (β), 95% confidence intervals (CI), and standardized (std.) 95% CI. Additionally, the within-person variance (σ^2^), the between-person variance (τ_00_ _family_), the intraclass correlation coefficient (ICC), the number of families (N _family_), the number of observations, and the marginal and conditional R² are displayed.

|  | **Global Physical Activity Questionnaire**  **MVPA/week** | | | | |
| --- | --- | --- | --- | --- | --- |
| *Predictors* | *Estimates* | *β* | *CI* | *std. CI* | *p* |
| (Intercept) | -305.25 | -0.14 | -1148.94 – 538.45 | -0.32 – 0.04 | 0.478 |
| group | -9.82 | -0.00 | -330.34 – 310.70 | -0.17 – 0.16 | 0.952 |
| timepoint [T1] | 116.87 | 0.12 | -135.75 – 369.48 | -0.06 – 0.29 | 0.364 |
| timepoint [T2] | 423.13 | 0.26 | 142.83 – 703.44 | 0.07 – 0.44 | **0.003** |
| self-efficacy PA | 41.57 | 0.13 | 11.55 – 71.59 | 0.04 – 0.23 | **0.007** |
| M intrinsic PA | -0.30 | -0.00 | -7.06 – 6.45 | -0.11 – 0.10 | 0.930 |
| family health climate PA | 26.24 | 0.20 | 11.80 – 40.68 | 0.09 – 0.32 | **<0.001** |
| intention PA | -103.26 | -0.10 | -195.32 – -11.20 | -0.20 – -0.01 | **0.028** |
| intention app PA | 3.10 | 0.00 | -60.03 – 66.22 | -0.08 – 0.09 | 0.923 |
| health | -5.73 | -0.00 | -106.02 – 94.57 | -0.09 – 0.08 | 0.911 |
| Sex [1] | 46.36 | 0.05 | -114.81 – 207.52 | -0.12 – 0.21 | 0.572 |
| adult child | -199.66 | -0.10 | -355.46 – -43.86 | -0.18 – -0.02 | **0.012** |
| group × timepoint [T1] | -4.88 | -0.00 | -355.86 – 346.09 | -0.18 – 0.17 | 0.978 |
| group × timepoint [T2] | -314.93 | -0.16 | -689.38 – 59.51 | -0.35 – 0.03 | 0.099 |
| **Random Effects** | | | | | |
| σ^2^ | 708377.58 | | | | |
| τ_00_ _family_ | 141594.53 | | | | |
| ICC | 0.17 | | | | |
| N _family_ | 52 | | | | |
| Observations | 513 | | | | |
| Marginal R^2^ / Conditional R^2^ | 0.132 / 0.276 | | | | |

**Table S2.** Linear mixed model analysis for the influence of the three week intervention period on device-based measured physical activity (accelerometry using 10 second epochs) in adults and children. Displayed are the results of the group (control = 0, intervention = 1) x time (dummy coded with T_0_ as reference for T_1_) interaction, and the secondary outcomes self-efficacy, intrinsic motivation, the family health climate, intention for behavior change, intention towards app use for behavior change, (all relating to physical activity), and health status as control variables. Additionally, adult/child (0 = adult, 1 = child), sex (0 = male, 1 = female), and non wear time (nwt) per week are included as a control variables. All results are displayed using the raw estimates (minutes of moderate to vigorous physical activity (MVPA) per week), the standardized Beta (β), 95% confidence intervals (CI), and standardized (std.) 95% CI. Additionally, the within-person variance (σ^2^), the between-person variance (τ_00_ _family_), the intraclass correlation coefficient (ICC), the number of families (N _family_), the number of observations, and the marginal and conditional R² are displayed.

|  | **Accelerometry**  **MVPA/week** | | | | |
| --- | --- | --- | --- | --- | --- |
| *Predictors* | *Estimates* | *β* | *CI* | *std. CI* | *p* |
| (Intercept) | 453.60 | -0.38 | 159.38 – 747.81 | -0.66 – -0.11 | **0.003** |
| group [intervention] | 27.77 | 0.10 | -64.35 – 119.90 | -0.23 – 0.43 | 0.553 |
| timepoint [T1] | 61.66 | 0.22 | -13.77 – 137.09 | -0.05 – 0.49 | 0.109 |
| self-efficacy PA | 8.88 | 0.10 | -2.08 – 19.83 | -0.02 – 0.23 | 0.112 |
| M intrinsic PA | 0.35 | 0.02 | -2.11 – 2.82 | -0.11 – 0.15 | 0.779 |
| FHC PA | -0.66 | -0.02 | -5.57 – 4.24 | -0.15 – 0.11 | 0.790 |
| int PA | -50.49 | -0.19 | -82.58 – -18.40 | -0.31 – -0.07 | **0.002** |
| int app PA | -5.17 | -0.02 | -28.30 – 17.96 | -0.14 – 0.09 | 0.660 |
| health | 20.77 | 0.07 | -13.68 – 55.22 | -0.04 – 0.18 | 0.236 |
| adult child [adult] | 9.61 | 0.03 | -47.67 – 66.90 | -0.17 – 0.24 | 0.741 |
| Sex [1] | 164.98 | 0.59 | 108.37 – 221.60 | 0.39 – 0.79 | **<0.001** |
| group [intervention] × timepoint [T1] | -117.93 | -0.42 | -226.10 – -9.77 | -0.81 – -0.03 | **0.033** |
| **Random Effects** | | | | | |
| σ^2^ | 54907.21 | | | | |
| τ_00_ _family_ | 10248.96 | | | | |
| ICC | 0.16 | | | | |
| N _family_ | 52 | | | | |
| Observations | 310 | | | | |
| Marginal R^2^ / Conditional R^2^ | 0.179 / 0.308 | | | | |

**Table S3.** Linear mixed model analysis for the influence of the three week intervention period on device-based measured physical activity (accelerometry using 10 second epochs) in adults and children. Displayed are the results of the group (control = 0, intervention = 1) x time (dummy coded with T_0_ as reference for T_1_) interaction, and the secondary outcomes self-efficacy, intrinsic motivation, the family health climate, intention for behavior change, intention towards app use for behavior change, (all relating to physical activity), and health status as control variables. Additionally, adult/child (0 = adult, 1 = child), sex (0 = male, 1 = female), and non wear time (nwt) per week are included as a control variables. All results are displayed using the raw estimates (step count per week), the standardized Beta (β), 95% confidence intervals (CI), and standardized (std.) 95% CI. Additionally, the within-person variance (σ^2^), the between-person variance (τ_00_ _family_), the intraclass correlation coefficient (ICC), the number of families (N _family_), the number of observations, and the marginal and conditional R² are displayed.

|  | **Accelerometry**  **step count/week** | | | | |
| --- | --- | --- | --- | --- | --- |
| *Predictors* | *Estimates* | *β* | *CI* | *std. CI* | *p* |
| (Intercept) | 68893.06 | 0.11 | 45554.62 – 92231.51 | -0.18 – 0.40 | **<0.001** |
| group [intervention] | 3224.60 | 0.15 | -4348.68 – 10797.87 | -0.20 – 0.50 | 0.403 |
| timepoint [T1] | 2621.34 | 0.12 | -3108.66 – 8351.34 | -0.14 – 0.39 | 0.369 |
| self-efficacy PA | -86.75 | -0.01 | -935.02 – 761.53 | -0.14 – 0.11 | 0.841 |
| M intrinsic PA | -116.87 | -0.08 | -307.18 – 73.44 | -0.21 – 0.05 | 0.228 |
| FHC PA | 209.00 | 0.07 | -183.13 – 601.13 | -0.06 – 0.21 | 0.295 |
| int PA | -5785.53 | -0.28 | -8264.07 – -3306.99 | -0.40 – -0.16 | **<0.001** |
| int app PA | -1773.42 | -0.11 | -3557.67 – 10.84 | -0.22 – 0.00 | 0.051 |
| health | 1341.20 | 0.06 | -1323.80 – 4006.20 | -0.06 – 0.17 | 0.323 |
| adult child [adult] | -6048.59 | -0.28 | -10424.08 – -1673.10 | -0.49 – -0.08 | **0.007** |
| Sex [1] | -447.16 | -0.02 | -4777.99 – 3883.67 | -0.22 – 0.18 | 0.839 |
| group [intervention] × timepoint [T1] | -8151.52 | -0.38 | -16378.62 – 75.57 | -0.76 – 0.00 | 0.052 |
| **Random Effects** | | | | | |
| σ^2^ | 315635210.49 | | | | |
| τ_00_ _family_ | 86440379.09 | | | | |
| ICC | 0.21 | | | | |
| N _family_ | 52 | | | | |
| Observations | 310 | | | | |
| Marginal R^2^ / Conditional R^2^ | 0.126 / 0.314 | | | | |

**Table S4.** Linear mixed model analysis for the influence of the three week intervention period on self-reported fruit and vegetable intake (single item questionnaire) in adults and children. Displayed are the results of the group (control = 0, intervention = 1) x time (dummy coded with T_0_ as reference for T_1_ and T_2_) interaction, and the secondary outcomes self-efficacy, intrinsic motivation, the family health climate, intention for behavior change, intention towards app use for behavior change, (all relating to healthy eating), and health status as control variables. Additionally, adult/child (0 = adult, 1 = child), and sex (0 = male, 1 = female) are included as a control variables. All results are displayed using the raw estimates (fruit and vegetable portions per week), the standardized Beta (β), 95% confidence intervals (CI), and standardized (std.) 95% CI. Additionally, the within-person variance (σ^2^), the between-person variance (τ_00_ _family_), the intraclass correlation coefficient (ICC), the number of families (N _family_), the number of observations, and the marginal and conditional R² are displayed.

|  | **Questionnaire**  **fruit and vegetable intake/week** | | | | |
| --- | --- | --- | --- | --- | --- |
| *Predictors* | *Estimates* | *β* | *CI* | *std. CI* | *p* |
| (Intercept) | -7.64 | 0.05 | -16.43 – 1.15 | -0.13 – 0.22 | 0.089 |
| group | 1.37 | 0.06 | -2.42 – 5.16 | -0.10 – 0.22 | 0.477 |
| timepoint [T1] | 0.57 | 0.11 | -1.97 – 3.11 | -0.04 – 0.26 | 0.660 |
| timepoint [T2] | 0.31 | -0.05 | -2.43 – 3.06 | -0.20 – 0.11 | 0.823 |
| self-efficacy NU | 0.66 | 0.19 | 0.40 – 0.91 | 0.11 – 0.26 | **<0.001** |
| M intrinsic NU | 0.05 | 0.08 | -0.00 – 0.10 | -0.00 – 0.17 | 0.061 |
| FHC NU | 0.11 | 0.07 | -0.04 – 0.26 | -0.03 – 0.17 | 0.157 |
| int NU | 4.25 | 0.30 | 3.04 – 5.46 | 0.22 – 0.39 | **<0.001** |
| int app NU | -0.73 | -0.08 | -1.46 – 0.00 | -0.16 – 0.00 | 0.051 |
| health | 0.69 | 0.05 | -0.33 – 1.72 | -0.02 – 0.12 | 0.184 |
| adult child | -2.04 | -0.09 | -3.62 – -0.46 | -0.15 – -0.02 | **0.012** |
| Sex [1] | -2.03 | -0.17 | -3.68 – -0.38 | -0.31 – -0.03 | **0.016** |
| group × timepoint [T1] | 1.42 | 0.06 | -2.07 – 4.91 | -0.09 – 0.21 | 0.425 |
| group × timepoint [T2] | -1.64 | -0.07 | -5.37 – 2.09 | -0.23 – 0.09 | 0.387 |
| **Random Effects** | | | | | |
| σ^2^ | 71.75 | | | | |
| τ_00_ _family_ | 27.91 | | | | |
| ICC | 0.28 | | | | |
| N _family_ | 52 | | | | |
| Observations | 525 | | | | |
| Marginal R^2^ / Conditional R^2^ | 0.255 / 0.464 | | | | |

**Table S5.** Linear mixed model analysis for the influence of the three week intervention period on self-reported fruit and vegetable intake (diary) in adults and children. Displayed are the results of the group (control = 0, intervention = 1) x time (dummy coded with T_0_ as reference for T_1_) interaction, and the secondary outcomes self-efficacy, intrinsic motivation, the family health climate, intention for behavior change, intention towards app use for behavior change (all relating to healthy eating), and health status as control variables. Additionally, adult/child (0 = adult, 1 = child), and sex (0 = male, 1 = female) are included as control variables. All results are displayed using the raw estimates (fruit and vegetable portions per week), the standardized Beta (β), 95% confidence intervals (CI), and standardized (std.) 95% CI. Additionally, the within-person variance (σ^2^), the between-person variance (τ_00_ _family_), the intraclass correlation coefficient (ICC), the number of families (N _family_), the number of observations, and the marginal and conditional R² are displayed.

|  | **Diary**  **fruit and vegetable intake/week** | | | | |
| --- | --- | --- | --- | --- | --- |
| *Predictors* | *Estimates* | *β* | *CI* | *std. CI* | *p* |
| (Intercept) | 4.22 | 0.03 | -6.11 – 14.54 | -0.23 – 0.29 | 0.422 |
| group [intervention] | -0.11 | -0.01 | -4.18 – 3.96 | -0.35 – 0.33 | 0.958 |
| timepoint [T1] | -0.63 | -0.05 | -3.12 – 1.86 | -0.26 – 0.15 | 0.617 |
| self-efficacy NU | 0.29 | 0.08 | -0.01 – 0.60 | -0.00 – 0.17 | 0.055 |
| M intrinsic NU | 0.11 | 0.17 | 0.05 – 0.17 | 0.07 – 0.26 | **0.001** |
| FHC NU | 0.02 | 0.02 | -0.15 – 0.20 | -0.10 – 0.13 | 0.804 |
| int NU | 3.61 | 0.26 | 2.22 – 5.00 | 0.16 – 0.36 | **<0.001** |
| int app NU | 0.22 | 0.02 | -0.63 – 1.07 | -0.07 – 0.11 | 0.617 |
| health | 0.11 | 0.01 | -1.00 – 1.22 | -0.07 – 0.09 | 0.845 |
| Sex [1] | -2.45 | -0.20 | -4.37 – -0.53 | -0.36 – -0.04 | **0.013** |
| group [intervention] × timepoint [T1] | 4.17 | 0.35 | 0.74 – 7.60 | 0.06 – 0.63 | **0.017** |
| **Random Effects** | | | | | |
| σ^2^ | 68.51 | | | | |
| τ_00_ _family_ | 35.74 | | | | |
| ICC | 0.34 | | | | |
| N _family_ | 52 | | | | |
| Observations | 371 | | | | |
| Marginal R^2^ / Conditional R^2^ | 0.228 / 0.493 | | | | |

**Table S6.** Linear mixed model analysis for the influence of the three week intervention period on self-reported joint physical activities per week in the families. Displayed are the results of the group (control = 0, intervention = 1) x time (dummy coded with T_0_ as reference for T_1_ and T_2_) interaction, and the control variable adult/child (0 = adult, 1 = child). All results are displayed using the raw estimates (number of joint physical activities per week), the standardized Beta (β), 95% confidence intervals (CI), and standardized (std.) 95% CI. Additionally, the within-person variance (σ^2^), the between-person variance (τ_00_ _family_), the intraclass correlation coefficient (ICC), the number of families (N _family_), the number of observations, and the marginal and conditional R² are displayed.

|  | **com_PA/Week** | | | | | |
| --- | --- | --- | --- | --- | --- | --- |
| *Predictors* | *Estimates* | *std. Beta* | *CI* | *standardized CI* | *p* | *std. p* |
| (Intercept) | 2.03 | -0.33 | 0.97 – 3.10 | -0.53 – -0.13 | **<0.001** | **0.001** |
| group | -1.08 | -0.15 | -2.52 – 0.36 | -0.35 – 0.05 | 0.142 | 0.142 |
| timepoint [T1] | 0.91 | 0.44 | 0.12 – 1.71 | 0.28 – 0.60 | **0.025** | **<0.001** |
| timepoint [T2] | 3.29 | 1.20 | 2.18 – 4.40 | 0.99 – 1.41 | **<0.001** | **<0.001** |
| adult child | 0.02 | 0.00 | -0.47 – 0.51 | -0.06 – 0.07 | 0.928 | 0.928 |
| group × timepoint [T1] | 1.31 | 0.18 | 0.15 – 2.48 | 0.02 – 0.34 | **0.027** | **0.027** |
| group × timepoint [T2] | 2.07 | 0.29 | 0.53 – 3.62 | 0.07 – 0.50 | **0.008** | **0.008** |
| **Random Effects** | | | | | | |
| σ^2^ | 5.76 | | | | | |
| τ_00_ _family_ | 5.13 | | | | | |
| ICC | 0.47 | | | | | |
| N _family_ | 50 | | | | | |
| Observations | 379 | | | | | |
| Marginal R^2^ / Conditional R^2^ | 0.195 / 0.575 | | | | | |

**Table S7.** Linear mixed model analysis for the influence of the three week intervention period on self-reported joint meals per week in the families. Displayed are the results of the group (control = 0, intervention = 1) x time (dummy coded with T_0_ as reference for T_1_ and T_2_) interaction, and the control variable adult/child (0 = adult, 1 = child). All results are displayed using the raw estimates (number of joint meals per week), the standardized Beta (β), 95% confidence intervals (CI), and standardized (std.) 95% CI. Additionally, the within-person variance (σ^2^), the between-person variance (τ_00_ _family_), the intraclass correlation coefficient (ICC), the number of families (N _family_), the number of observations, and the marginal and conditional R² are displayed.

|  | **com_NU/Week** | | | | | |
| --- | --- | --- | --- | --- | --- | --- |
| *Predictors* | *Estimates* | *std. Beta* | *CI* | *standardized CI* | *p* | *std. p* |
| (Intercept) | 8.47 | -0.18 | 6.91 – 10.03 | -0.42 – 0.06 | **<0.001** | 0.142 |
| group | -0.28 | -0.03 | -2.43 – 1.87 | -0.27 – 0.21 | 0.797 | 0.797 |
| timepoint [T1] | 0.33 | 0.21 | -0.27 – 0.92 | 0.12 – 0.30 | 0.280 | **<0.001** |
| timepoint [T2] | 4.70 | 0.69 | 3.89 – 5.51 | 0.56 – 0.83 | **<0.001** | **<0.001** |
| adult child | 0.00 | 0.00 | -0.31 – 0.32 | -0.03 – 0.04 | 0.984 | 0.984 |
| group × timepoint [T1] | 1.22 | 0.14 | 0.40 – 2.04 | 0.04 – 0.23 | **0.004** | **0.004** |
| group × timepoint [T2] | -3.07 | -0.34 | -4.26 – -1.87 | -0.47 – -0.21 | **<0.001** | **<0.001** |
| **Random Effects** | | | | | | |
| σ^2^ | 2.01 | | | | | |
| τ_00_ _family_ | 14.31 | | | | | |
| ICC | 0.88 | | | | | |
| N _family_ | 50 | | | | | |
| Observations | 319 | | | | | |
| Marginal R^2^ / Conditional R^2^ | 0.090 / 0.888 | | | | | |
